# Using design research and human-centered design to address growing pains in a busy, urban emergency department: a faculty, clinician, and student collaboration between nursing, design, and medicine

**DOI:** 10.1186/s12245-024-00586-5

**Published:** 2024-02-05

**Authors:** Mikael L. Avery, Jacquelyn L. Arena, Nicholas D. Benson, Neil A. Ray, Marion Leary

**Affiliations:** 1 University of Pennsylvania, Philadelphia, PA USA; 2 Hospital of the University of Pennsylvania, Philadelphia, PA USA

**Keywords:** Design thinking, Human-centered design, Emergency medicine, Materials management, Design, Emergency department operations

## Abstract

In 2021, a large urban university-based hospital transitioned to a new two-floor emergency department. Despite the new environment, there were usability and workflow challenges with the space. The authors of this paper created a multidisciplinary, human-centered design collaborative of clinicians, university faculty, and students in an effort to increase emergency department efficiency. After thorough design-research and clinician-focused collaboration, the authors and design team identified the need to improve medical supply retrieval time, which directly impacts patient care and clinician satisfaction. The primary interventions consisted of a redesign that is as follows: (a) created standardized icons related to organ system, (b) increased visibility of supply labels, and (c) reorganized supplies based on usage data. Although a successful project, it was not without several barriers discussed in this article, including design researcher and clinician-level setting and engagement, academic/institutional policies, and conflicting schedules. In addition, the lessons learned from implementing human-centered design concepts into clinical workflow sets forth future research opportunities and inspiration for other institutions to collaborate.

## Background

The University of Pennsylvania School of Nursing (Penn Nursing) and the Weitzman School of Design (School of Design) collaborated on a project with the Hospital of the University of Pennsylvania’s (HUP) new two-story emergency department (ED). Shortly after the new emergency department opened in 2021, the ED staff and clinicians began noticing design and usability issues.

The ED Pavilion Design Thinking Project was an opportunity for students to collaborate across the university to bring human-centered design (HCD) and design thinking (DT) processes into practice in a clinical environment—working with ED providers to identify needs and develop solutions. HCD is an approach of designing solutions that centers the needs of the individuals experiencing the problem [1]. Design thinking is a five-step process, used to work through the HCD approach, which focuses on empathizing with the end users, defining the problem from their point of view, and then ideating, prototyping, and testing the solution [2, 3]. As noted by Roberts [4], “Design thinking is not a checklist of protocols, instead it is a translatable practice framework that can be learned and embedded within the DNA of an organization.” Design thinking fundamentally diverges from the traditional scientific method, which is more familiar in the healthcare environment. The scientific method starts with a pre-formed hypothesis, leading to a single intervention, which is subsequently tested through direct inquiry during extensive experiments. In contrast, design thinking begins by deconstructing a problem, understanding the problem space, generating a series of potential solutions, and prototyping the best solutions. The process does not start with a premeditative intervention but dedicates time to understanding the problem fully before proposing a solution. In the healthcare environment, while those trained with a science background often emphasize the analysis of pre-formed hypotheses, the design professional brings together diverse sources to create new and unconventional solutions [4]. This perceived difference can initially be a barrier to adoption of DT techniques, but as this case study demonstrates, through implementing the DT process, including co-design with clinicians, DT and HCD practices can achieve support and create a positive contribution within the healthcare setting. As a result of this initiative, the design team proposed innovative solutions for materials management. By making medical supplies easier to find and more accessible, the team anticipated a positive impacting on patient care.

### Practice innovation

The HUP ED is one of the few hospitals in the country to feature an emergency department that spans two floors. To reduce the scale of the ED’s large footprint, patient acuity is used as a metric to organize and divide the space into different silos. The resulting zones are therefore intended to make the space feel smaller. However, issues emerged as the practitioners began to occupy and treat patients within the new space.

Through a series of broad conversations around the use of HCD in healthcare between Penn Medicine Faculty, Penn Nursing, and the School of Design, an initiative was developed to engage students in the DT process to develop novel solutions for the challenges faced by emergency medicine providers.

Two undergraduate nursing and two graduate design students with experience in DT were chosen to participate. The clinical and faculty leads worked together to determine the timeline, milestones, deliverables, and expectations for the project. The project specifications were largely determined by the academic semester for students but also in collaboration with the emergency department leadership to produce meaningful and actionable deliverables.

To deploy a full DT experience that was structured on the human-centered process, students completed the following milestones. First, students received and reviewed comprehensive onboarding material that was developed specifically for this project. This document introduced terminology that was salient to emergency medicine, discussed patient flow through the department, and defined different staff roles. The goal of this document was to allow students a brief primer into the department, granting familiarity with departmental operations and allowing them to productively utilize their shadowing experiences. After reviewing the onboarding document, the students then shadowed ED clinicians, including both nurses and physicians, over three separate observation shifts lasting 4-h each. These shadowing experiences included at least one design and one nursing student. A pair of students, again consisting of one design and one nursing student, then conducted eight end-user interviews of ED staff. These interviews were conducted outside of clinical hours with a variety of stakeholders, including nurses, physicians, pharmacists, advanced practice providers, and ED technicians. The interviewees were chosen on a volunteer basis and included people who were interested in learning more about the design thinking process. In addition to these formal interviews, the observation sessions provided opportunities for more organic interactions and input. Weekly check-ins were conducted with Penn Nursing and the School of Design faculty. The team shared insights with ED leadership in midpoint and final presentations.

**Fig. 1:**
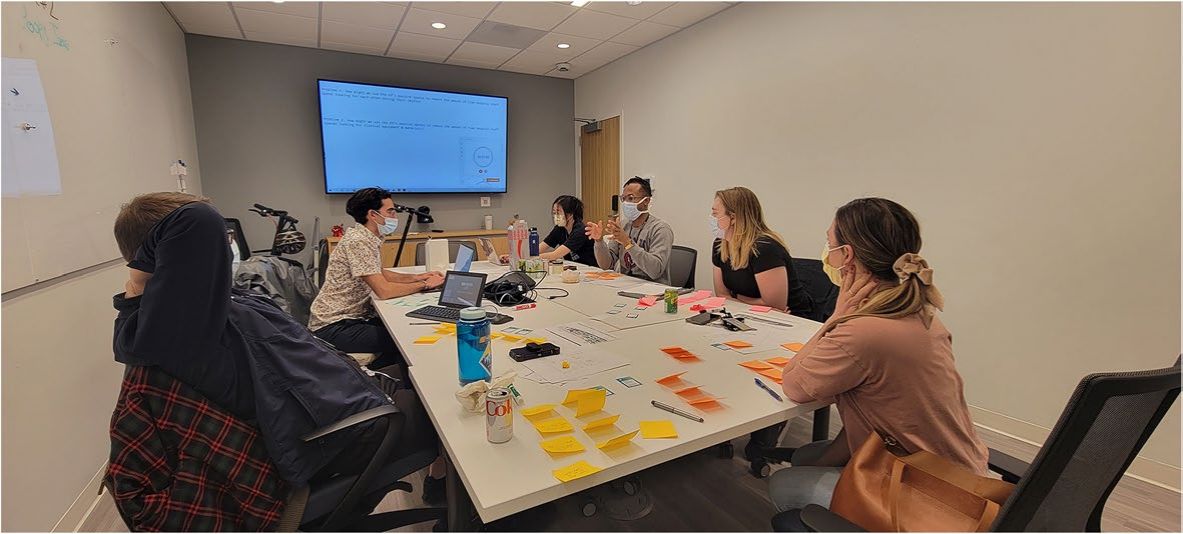
One co-design session held at the ED

As faculty met with students, iterative improvements to the project structure were implemented, a reflection of the nature of HCD research. These adjustments included adding a third 4-h shift per student, two in-person co-design sessions in the ED with interested staff and clinicians (Fig. 1), and design “office hours” where students completed their analysis and synthesis work on-site allowing for real-time feedback and discussion with clinicians.

These improvements were implemented to achieve design goals and in response to both student and clinician needs. For example, increasing the number of observational sessions granted the students more time in the ED and additional timeslots, allowing them to better align schedules and observe as a team. The on-site office hours and co-design sessions were added in response to the need for more access to clinical spaces and clinical staff input. The co-design sessions were hands-on and engaging, requiring clinicians’ full attention. In addition to allowing for the rapid exploration and development of various project paths, the co-design sessions also provided an opportunity for clinicians to experience, firsthand, the design process and its methods. These sessions were complemented by in-house office hours, which allowed for more organic and ad hoc engagement. The value of these real-time changes was to increase clinical staff engagement and to provide more hands-on opportunities for the students to trial their ideas. This was crucial, because it increased trust in the design team and allowed clinicians to be more candid in their opinions. It demonstrated investment by the design team in the problem and reciprocal investment by the staff in the DT process.

Working through the design process as outlined, the team identified a well-defined and targeted research question: Can a multidisciplinary human-centered design approach address the inefficiencies associated with medical supply retrieval and positively impact the subjective frustration experienced by emergency department employees? If addressed successfully, the team anticipated that this would have an outsized impact on department operations. As this problem was not an active area of focus for the department, the team had more creative space to work, free of precedent efforts. It was also a project that could tangibly be undertaken and completed in one semester (15 weeks). Furthermore, it was a problem space that is not routinely examined by typical hospital quality improvement efforts.

After collecting and analyzing initial data, it became clear that nurses and physicians were spending unsustainable amounts of time walking from supply room to supply room across distant zones of the ED, struggling to find the appropriate materials. Multiple providers reported that time spent away from direct patient care had the potential to pose a patient safety vulnerability. Two provider statements highlight this finding: “There will be times where I'm literally grabbing and throwing things onto the floor trying to find something” and “I feel like I'm spending way too much time standing in the room just trying to find one random item.” Not only were supplies stored in various locations (such as dedicated equipment rooms, hallways, or rooms shared by support staff) but also there was also a lack of standardization within individual shelves. Overall, this led to multiple issues including (a) increased frustration with supply organization, (b) workplace dissatisfaction, (c) challenges with onboarding new staff, and (d) adverse effects on team dynamics. These were recurrent anecdotal themes that came up during clinician interviews and were witnessed firsthand during observation periods.

As mentioned above, the HCD/DT processes incorporate defined, iterative steps (empathizing with stakeholders, defining the problem, ideating solutions, prototyping, and testing) with specific techniques employed at each step. To begin, interviews and observations created the context for empathy building as the design team saw and heard what factors impacted the lived experience of those working in the ED.

During the define phase, the team analyzed, processed, and then presented their findings to clinical staff and administrators, finding agreement that supply management was a key leverage point that could positively impact all stakeholders. The team then engaged in co-design sessions and on-site design office hours to better understand key aspects of supply management and identify possible solutions, ultimately focusing on time to locate supplies, the pain point anticipated to have the greatest impact on provider frustration. Bringing all this work together, the team created testable mock-ups of the proposed solution in the ED, allowed for testing and learning through continued engagement with clinicians.

### The solution

Leveraging both internal design sessions and co-creation with clinical staff, the team developed a solution that hinged on three interconnected elements.

**Fig. 2:**
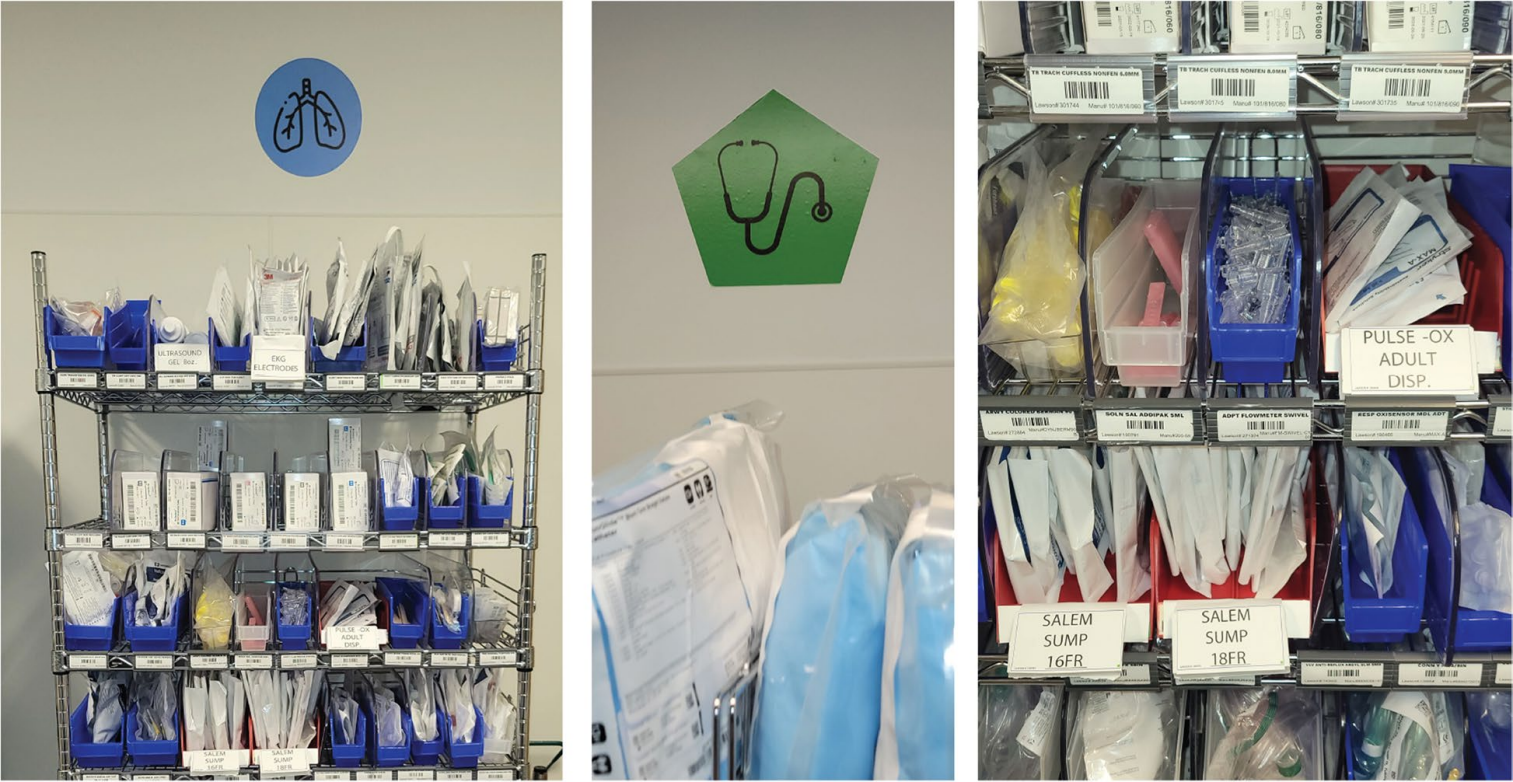
Vinyl sticker prototypes and modified item labeling approach implemented in the ED

The first was to organize the materials based on body systems. Supply shelves were designated with vinyl stickers indicating the relevant organ system, such as respiratory, gastrointestinal, and cardiac. For example, bronchodilators would be found on the respiratory shelf (Fig. 2). To maximize identifiability, each vinyl sticker had a unique color schema, shape, and symbol. This schema would translate across supply rooms, allowing clinicians to quickly locate supplies even when working in a different physical space.

**Fig. 3:**
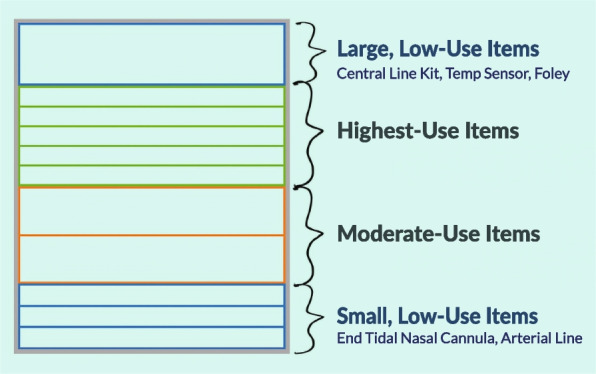
Material storage strategy to increase consistency across the ED

The second solution was to increase consistency between material storage areas. More frequently used, smaller items will be stocked at waist height (making it more visually apparent and ergonomic), and less frequently used items would be placed on the top or bottom shelves (Fig. 3). Additionally, increasing the visibility of supply labels through larger fonts and distinct colors would make it easier to obtain the necessary materials expeditiously.

Finally, the team recommended using material usage data to optimize storage across shelves. Data analysis showed that just 15 items represented 30% of all material usage, while 52% of all unique items only represented 3% of all material usage. In monetary terms, the team identified US $13,300 worth of materials that were not touched in the previous 6 months. Despite this, these supplies were stocked in equal quantities. Additionally, low-use items were stocked next to high-use items, making the shelves much more cluttered. To address this, the team determined that items needed once per week or less could safely be placed in less prime locations while using the previously detailed identifiability strategies to ensure that they could be located quickly and easily. This data-driven approach brought a unique perspective to materials management decision-making and has broad implications for patient care workflows. A cursory analysis of provider survey data (*n* = 10) showed a decrease in stress or difficulty locating supplies. On a Likert scale, with 0 being the least stressful or difficult and 5 being the most stressful or difficult, stress decreased from 3.6 to 2.7, and difficulty decreased from 3.9 to 1.9.

At the conclusion of the project, the team delivered a formal design proposal with visual presentation to ED clinicians and leadership, including the department medical director and chair. While all aspects of the solution were well received, the item usage data elicited the strongest positive feedback, in part, because there was a tangible financial metric associated with it. Leadership was receptive to the idea that substantial department funding was being spent on supplies that were not routinely used. Most importantly, department leadership found that the identified problem and proposed solutions aligned with current department goals, such as increasing ED efficiency and throughput. ED leadership enthusiastically received the final recommendations. Following additional alignment with ED best practices and guidelines, many of the recommendations will go into effect over the coming months.

### Student and clinical impact

A key moment occurred early in the process, while the clinical team members provided the students with an extended tour of the ED. Having both student groups tour simultaneously provided them immediate learning through one another’s questions and observations pertaining to the physical, social, and medical environment. One of the School of Design students stated, “I love working with content experts on the team,” as he noted that the Penn Nursing student asked questions that he may have taken days to see as relevant, if at all. By bringing these two groups together on the same team, the number of collective blind spots decreased dramatically. Furthermore, over the course of the project, when questions arose that were outside the scope of their training (e.g., healthcare protocols and operations or a potential design—research technique, or activity), answers could be provided instantaneously and from within the team.

The School of Design students’ expertise had similar impacts on the clinical team. As noted by one clinical team member, a physician’s perspective on department design is likely to be incomplete, overly focused on a single discipline’s needs, and trained by experience to stay within the bounds of what is perceived as standard or necessary. By partnering with students trained in design, clinicians are inviting an outsider’s perspective to help access unique and creative solutions. Furthermore, by working with nurses and nursing students, physicians on the team are fostering collaborative relationships and expanding knowledge of colleagues’ responsibilities, challenges, and needs. The impact of these experiences extends beyond the active project.

Early in the process, some members of the clinical staff expressed concern that students from outside of the ED lacked key understanding and had little power to enact change, and this perception could have impacted initial research stages. However, following the first co-design sessions, this sentiment began to change. Once the clinical staff saw firsthand how interested the students were in learning and how effectively they shaped the project’s direction, they expressed more positive sentiments toward the research and started finding more opportunities to engage with students.

Beginning with staff interviews and observations is a well-established approach in DT. However, the early stages of this project highlighted the potential of an alternative approach. In an environment full of people trained to act and steeped in discipline-specific knowledge, engaging them on their terms (through action), and bringing their expertise to bear through activity, can open doors and forge stronger partnerships. This can be achieved through talk-aloud observations and similar approaches. However, these tactics are not always well-suited to the bustling environment and patient privacy needs of the ED. In future iterations of this work, the team plans to build in early co-research sessions that respect the unique environment of the ED. This has the potential to help researchers acquire deeper knowledge and to create more opportunities for clinicians to contribute through action.

## Conclusion

The interdisciplinary collaboration brought about by this design-research project proved beneficial, both clinically and academically, to the ED workflow. This was highlighted in positive feedback by clinical staff during the final design implementation trial. Having developed a working relationship with design and nursing students during the design process, staff were eager to engage in trial implementation. While the designed solution was neither complex nor resource-intensive, it has the potential for great impact on department flow. Collaborators and stakeholders agreed that this would streamline workflows, speed up processes, and reduce potential errors.

As a result of this work, department leaders endorse the value of human-centered design, creating an opportunity to increase future stakeholders and establishing the potential for larger projects. Incorporating insights from this project will allow for new department design opportunities and early identification of barriers. This work exemplifies the benefits of interdisciplinary collaboration between students and clinicians, allowing us to rethink teaching and innovation in emergency medicine.

## Data Availability

Data sharing is not applicable to this article as no datasets were generated or analyzed during the current study.

## Abbreviations

HUP: Hospital of the University of Pennsylvania
ED: Emergency department
HCD: Human-centered design
DT: Design thinking
